# An integrative approach to predicting the functional effects of small indels in non-coding regions of the human genome

**DOI:** 10.1186/s12859-017-1862-y

**Published:** 2017-10-06

**Authors:** Michael Ferlaino, Mark F. Rogers, Hashem A. Shihab, Matthew Mort, David N. Cooper, Tom R. Gaunt, Colin Campbell

**Affiliations:** 1 Big Data Institute, University of Oxford, Oxford, OX3 7LF UK; 2 Nuffield Department of Obstetrics and Gynaecology, University of Oxford, Oxford, OX3 9DU UK; 3 Intelligent Systems Laboratory, University of Bristol, Bristol, BS8 1UB UK; 4 MRC Integrative Epidemiology Unit, University of Bristol, Bristol, BS8 2BN UK; 5 Institute of Medical Genetics, Cardiff University, Cardiff, CF14 4XN UK

**Keywords:** Indels, Non-coding genome, Variant prioritisation, Support vector machines

## Abstract

**Background:**

Small insertions and deletions (indels) have a significant influence in human disease and, in terms of frequency, they are second only to single nucleotide variants as pathogenic mutations. As the majority of mutations associated with complex traits are located outside the exome, it is crucial to investigate the potential pathogenic impact of indels in non-coding regions of the human genome.

**Results:**

We present FATHMM-indel, an integrative approach to predict the functional effect, pathogenic or neutral, of indels in non-coding regions of the human genome. Our method exploits various genomic annotations in addition to sequence data. When validated on benchmark data, FATHMM-indel significantly outperforms CADD and GAVIN, state of the art models in assessing the pathogenic impact of non-coding variants. FATHMM-indel is available via a web server at *indels.biocompute.org.uk*.

**Conclusions:**

FATHMM-indel can accurately predict the functional impact and prioritise small indels throughout the whole non-coding genome.

**Electronic supplementary material:**

The online version of this article (doi:10.1186/s12859-017-1862-y) contains supplementary material, which is available to authorized users.

## Background

The advent of next generation sequencing technologies has led to a rapid increase in identified genetic variation, including single nucleotide variants (SNVs), copy number variants, insertions and deletions (indels), in addition to larger scale DNA rearrangements. There are now a vast number of biomedical applications exploiting genomic sequence data and such data will play a crucial role in personalised medicine. As a consequence, interpretation of the functional impact of identified variants is of increasing importance. This has led to the development of accurate methods for assessing genomic tolerance and predictive techniques for discriminating between harmful (pathogenic) and neutral mutations [1–4].

In the past, there has been an emphasis on using sequencing technologies to study human exomes, rather than full genomes, owing to the reduced costs involved and a primary focus towards those regions of the genome deemed to be most functionally relevant. Accordingly, the vast majority of models for predicting the functional impact of indels have been restricted to their effect in the human exome – see e.g. [5–7].

However, the portion of the genome which codes for proteins accounts for only about 2% of the whole sequence, and it is becoming increasingly evident that non–coding portions of the genome play crucial functional roles in human development and disease [8]. For example, a germline deletion in the micro RNA MIR17HG leads to microcephaly [9], and a mutation in the promoter region of MIR146A is genetically associated with lupus [10]. Furthermore, most SNVs identified by genome wide association studies (GWASs) as correlated with increased risk of complex disease are located in non–coding regions [11].

Given examples like these, in this paper we focus on the association between non–coding variants and disease by developing a model for predicting the functional impact of indels in non–coding regions of the human genome. Our method can be seen as a generalisation of FATHMM [1] for prediction beyond point mutations. A web–based implementation of FATHMM–indel is available at *indels.biocompute.org.uk*.

## Methods

### Data collection

We developed a machine learning approach to classify the functional effects of small indels, that is, variants where the sequence change involves up to 20 base pairs. The term indel refers to micro insertions/deletions, i.e. mutations that either insert or delete a DNA string to the wildtype sequence.

Pathogenic non–coding indels were collected from the CinVar database [12]. From data downloaded on 8th January 2017, we extracted pathogenic mutations (clinical significance 5) *not* annotated as somatic. Neutral (likely benign) non–coding indels were collected from the exome variant server (EVS) data release ESP6500SIV2 [13]. We considered variants recorded in individuals of African ancestry since European and Asian populations have been subject to bottlenecks which might have resulted in pathogenic indels with relatively high minor allele frequencies (MAFs) – see e.g. [7]. Thus, to increase the probability that EVS mutations were truly benign polymorphisms, we only selected variants with MAF≥1*%* in individuals with African ancestry. In addition to using database annotations to collect micro insertions/deletions in non–coding regions, we further exploited Ensembl GRCh37 (release 85) annotations. By using annotated coding sequence regions, we were able to verify that all examples in our data sets did *not* fall within genomic regions annotated as protein coding.

Repeats are extremely challenging genomic elements to sequence as they are characterised by high sequencing error rates. For example, repeats are strongly affected by polymerase slippage which can potentially alter the length of the repetitive sequence mutation [14]. For these reasons, we conservatively filtered all repeats from our data sets. These steps combined yielded 2 523 pathogenic and 9 783 neutral examples.

### FATHMM-indel’s features

We used a variety of data sources which potentially carry information about an indel’s pathogenic status. Previous work on SNVs has shown that the best predictive models exploit information about sequence conservation in the vicinity of a mutation [2, 15]. Intuitively this makes sense as we expect that mutations occurring in highly conserved regions of a genome are more likely to have deleterious impact compared to those that occur in evolutionary variable regions. However, conservation metrics used to evaluate SNVs are based on distinct nucleotide positions within the human genome [1, 16, 17]. Hence, to study small indels, we must either revise these methods to produce conservation scores for longer ranges, or devise a method that uses existing single–nucleotide scores. Here we adopted the latter approach: to obtain conservation features for small regions, we treated each insertion or deletion as a series of mutations in the reference sequence. All features are described in details in the Additional file 1 (Supplementary Materials).

### FATHMM-indel’s model

We used a support vector machine (SVM) [18, 19] as our binary classifier, as SVMs have produced highly accurate classifiers for a variety of bioinformatics domains – see, e.g. [2, 15, 20, 21]. Kernel methods such as SVMs can easily handle structured data, such as strings and graphs, which are abundant in bioinformatic applications. Furthermore, support vector machines allow straightforward integration of heterogeneous biological data.

SVMs use kernel matrices to encode the similarity of data objects. Kernels have been derived for a number of different object types, from continuous and discrete variables, through to graph and sequence data (see e.g. [18] for an overview). In this work, we used a Gaussian kernel with precision *γ* and a “cost” parameter *C* to lessen the influence of noise in the data.

SVMs can be used to prioritise variants using Platt scaling [22]. Given a test instance **z**, SVMs compute an “uncalibrated” score

1 $$\begin{array}{@{}rcl@{}} f(\mathbf{z})=\sum_{i=1}^{N}\,\alpha_{i}\,y_{i}\,K(\mathbf{x}_{i}, \mathbf{z}) + b \end{array} $$

**K** represents the kernel matrix encoding the similarity between data points. The dual parameters *α*_*i*_ (Lagrange multipliers) and *b* (bias) are learned from training data. The sum in (1) runs over all training examples **x**_*i*_ with class labels *y*_*i*_=±1. The score *f*(**z**) can be interpreted as a confidence measure since, the larger the modulus | *f*(**z**) |, the greater the confidence of the prediction. *f*(**z**) can be converted into a standardised score *σ*(**z**)∈[0,1] by fitting of a logistic function

2 $$\begin{array}{@{}rcl@{}} \sigma(\mathbf{z})=\,\frac{1}{1+\text{exp}(A f(\mathbf{z})+B)} \end{array} $$

The parameters *A* and *B* are learned using maximum likelihood estimation on training data. Exploiting this approach, FATHMM–indel can prioritise variants by returning a score *σ* for each test mutation. A data point **z** is predicted as pathogenic (positive class) if *σ*(**z**)≥0.5 whilst it is predicted as neutral (negative class) otherwise. Indels with largest scores *σ* are the most likely to be pathogenic.

**Fig. 1:**
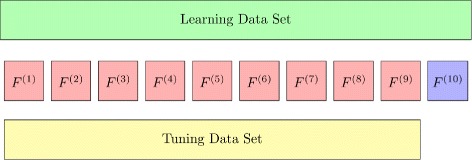
Nested cross validation. To implement nested cross validation, we split the data set into ten stratified folds. The figure shows *one* out of ten NCV loops. For each NCV iteration, an independent testing set (*F* ^(10)^ in the figure) is left out to assess FATHMM-indel’s performance. The remaining folds (red sets in the figure) are merged to create the tuning set used to learn, under cross validation, the optimal values of the hyperparameters. Crucially, a different fold is used as testing set in each iteration, fully exploiting all data to evaluate FATHMM-indel’s performance

The kernel machine we used is characterised by two hyperparameters (*C*,*γ*) that need to be optimised in order to select the best model to validate against currently published methods (see Results). One of the most popular protocols used for model selection is cross validation. However, it has empirically been shown that cross validation is susceptible of overfitting the *model selection* criterion and, consequently, provide an optimistic estimate of generalisation performance [23]. To control again this potential bias, we performed model selection using a rigorous nested cross validation (NCV) protocol. NCV is comprised of two (nested) loops of cross validation where the inner loop is used for hyperparameter tuning whilst the outer loop is used for performance assessment (Fig. 1). The data set is randomly split into ten *stratified* folds to ensure that each fold (approximately) contains the same number of examples for both classes. In each iteration of the outer loop, nine folds are used to create a tuning set whilst the remaining fold is used for testing. In the inner loop, a grid search is performed on the tuning set in order to select the optimal hyperparameters. A parameter space is created by setting up possible ranges for the hyperparameter values and an SVM is trained at each grid position in such space. The optimal model is selected by implementing ten–fold cross validation and accuracy is used as performance metric. Lastly, the best model is deployed on the testing set to assess the performance of the classifier. This procedure is repeated ten times (the number of stratified folds) and performance is evaluated using sensitivity, specificity, balanced accuracy, and area under the ROC curve (AUC).

## Results

### FATHMM-indel’s performance evaluation

The data collected are substantially imbalanced with many more neutral than pathogenic instances. Therefore, in order to annotate a balanced training set, it is necessary to subsample the majority (EVS) class. A data set can be created by selecting 2 523 pathogenic indels and randomly drawing 2 523 data points from EVS mutations. Using such a set, FATHMM–indel’s performance could be evaluated under nested cross validation.

However, it is crucial to establish whether our model is robust against subsampling of EVS mutations. Accordingly, we created 50 data sets comprised of 2 523 pathogenic and 2 523 randomly selected neutral indels. Our model was trained and tested on each set under nested cross validation. Performance was assessed by calculating averaged statistics and standard errors (SEs) across all 50 data sets. FATHMM–indel achieved an average performance of 89% sensitivity, 89% specificity, 89% balanced accuracy, and 0.95 AUC (Table 1). The small standard errors recorded show that our method is robust against subsampling of EVS indels.

**Table 1:** NCV experiment results. FATHMM-indel’s performance across 50 data sets created by randomly subsampling the neutral (EVS) class

Sensitivity (SE)	Specificity (SE)	Balanced accuracy (SE)	AUC (SE)
0.886 (0.005)	0.891 (0.005)	0.889 (0.004)	0.950 (0.003)

The small standard errors (SEs) indicate it is consistent to use one random EVS sample to train the final model

In the next section, we compare our model with published methods on benchmark data. The results from this section indicate FATHMM–indel’s performance is insensitive to subsampling of the neutral class. Therefore, to validate our method against state of the art models, we trained FATHMM–indel using a data set of 2 523 pathogenic and 2 523 randomly sampled neutral indels. The hyperparameters were set to the values which recorded highest balanced accuracy under nested cross validation experiments (*C*=10,*γ*=0.01).

### Validation against published methods

In this section we compare our method with CADD [2] and GAVIN [24] – two state of the art models for predicting the impact of non–coding indels. These methods allow comprehensive validation of FATHMM–indel as they are capable of assessing mutation tolerance throughout the *whole* non–coding genome (i.e. they are not restricted to specific units, e.g. splice sites).

CADD is a prioritisation tool capable of measuring deleteriousness by computing “*C* scores” for genetic variants. CADD’s ability to assess the functional impact of mutations was achieved by training an SVM to discriminate between fixed derived alleles in humans (depleted of deleterious variants) and simulated mutations (enriched with deleterious variants). CADD can also be used to classify the impact of mutations by selecting an optimal threshold for *C* scores. As suggested by CADD’s authors (through their model web server), all indels with scaled *C* scores of *at least* 15 were predicted as pathogenic.

In addition to predicting the functional class of mutations, FATHMM–indel can also prioritise each variant by computing a score *σ* (see Methods). For both CADD and FATHMM–indel, the higher the score, the higher the confidence the mutation is functional in disease.

GAVIN is a computational framework that, amongst others, exploits minor allele frequency data to calibrate its predictions. GAVIN does not rank mutations but only classifies the functional impact of a test indel as either pathogenic or neutral.

To perform an unbiased validation against CADD and GAVIN, we annotated a *balanced* benchmark data set comprised of mutations *not* used during the training of any model. Pathogenic indels were obtained from the human gene mutation database (HGMD) release 2014.v4 [25] whilst neutral instances comprised EVS indels with MAF≥1*%*. We restricted our validation examples to mutations that can be scored by all methods and, according to our data collection protocol, we did not consider variants located in repeat regions. Furthermore, we exploited database and Ensembl annotations to ensure all validation indels were *not* located in coding regions. This procedure yielded a benchmark data set with 853 pathogenic (HGMD) indels and 853 neutral (EVS) indels.

Performance was measured using sensitivity, specificity, balanced accuracy, and Matthews correlation coefficient (MCC). The results of our empirical validation on benchmark data are detailed in Table 2. FATHMM–indel recorded the best performance, achieving a balanced accuracy of 90% compared to 80% for CADD and 77% for GAVIN. The substantial improvement in performance attained by our model is also highlighted by the high MCC value, showing how FATHMM–indel’s predictions have the strongest correlation with the true class labels. Furthermore, the high sensitivity achieved by our model demonstrates FATHMM–indel’s ability to identify truly pathogenic variants. This underlines the potential practical usefulness of our model in, for example, clinical settings where it is crucial not to erroneously categorise pathogenic mutations. Both CADD and GAVIN manifest a bias towards assessing the impact of validation indels as neutral. This has allowed CADD and GAVIN to reach high specificities but very low sensitivities due to the high number of false negatives (FNs). GAVIN recorded the highest value of false negatives (FN=332, 39% of benchmark pathogenic indels), followed by CADD (FN=282), whereas FATHMM–indel is characterised by the lowest number FN=81 (9% of benchmark pathogenic indels). The somewhat lower specificity of our model is a consequence of a slightly higher false positive rate as 11% of benchmark neutral indels were erroneously predicted as pathogenic by FATHMM–indel, whilst 7% of validation neutral indels were miscategorised by CADD and GAVIN.

**Table 2:** Validation, on benchmark data, against published methods

	Sensitivity	Specificity	Balanced accuracy	MCC
FATHMM-indel	0.905	0.887	0.896	0.793
CADD	0.669	0.934	0.802	0.626
GAVIN	0.611	0.934	0.773	0.576

**Fig. 2:**
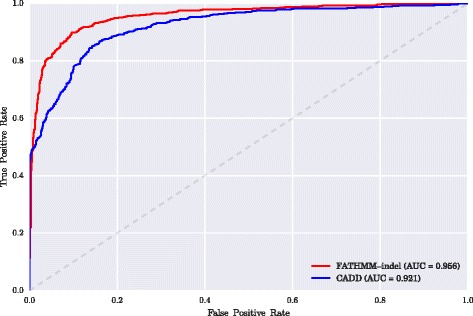
Empirical ROC curves for FATHMM-indel and CADD. Performance comparison, on benchmark data, between FATHMM-indel and CADD. ROC curves display sensitivities and false positive rates at all possible cutoff levels. Therefore, they can be used to assess the performance of a model independently of the decision threshold

Since both CADD and FATHMM–indel score variants for prioritisation, it is possible to further compare these models’ performance by means of ROC curves and corresponding AUC statistics. For binary classification, a ROC curve displays the true positive rate (sensitivity) as a function of the true negative rate (1−specificity). The points of the curve are computed by varying the decision threshold from the most positive (pathogenic) data point to the most negative (neutral) one. This allows us to comprehensively validate these models and analyse their performance over the range of possible classification thresholds. The area under the ROC curve, known as AUC, measures the ranking quality of a classification hypothesis [26]. A perfect classifier would have unit AUC whereas random guessing would achieve an AUC of 0.5. The ROC curves of FATHMM–indel and CADD, obtained using the benchmark data set, are visualised in Fig. 2. FATHMM–indel was the best performing method achieving an AUC of 0.956 compared to 0.921 of CADD.

In our validation experiments on benchmark data, FATHMM–indel has shown significant performance improvements over published models. This also validates the ability of FATHMM–indel to generalise to other data sets and establish FATHMM–indel scores as informative metrics for variant prioritisation.

### FATHMM-indel for population genetics

To further assess the validity of our approach, we collected non–coding indels from the latest data release (phase 3) of the 1 000 genomes (1KG) project [27]. Amongst its principal goals, the 1KG project aims at analysing the distribution of common and rare mutations in order to provide a broad representation of human genetic variation. In the project’s final phase (phase 3), 2 504 genomes were reconstructed from apparently healthy individuals which are stratified into the 5 “continental” populations of East Asia (EAS), South Asia (SAS), Europe (EUR), Africa (AFR), and America (AMR). The 1KG data set also annotates the allele frequency (AF) for each continental population as well as the allele frequency for the global (GLB) sample. This allows to comprehensively analyse private (population specific) alleles and shared variants.

By collecting small variants not located in repetitive regions, we were able to score 1,466,000 non–coding indels from 1KG data. FATHMM–indel classified the vast majority of mutations as neutral, achieving an accuracy of 96%. This represents additional evidence supporting the informativeness of FATHMM–indel’s scores for assessing genomic tolerance of non–coding variants.

**Fig. 3:**
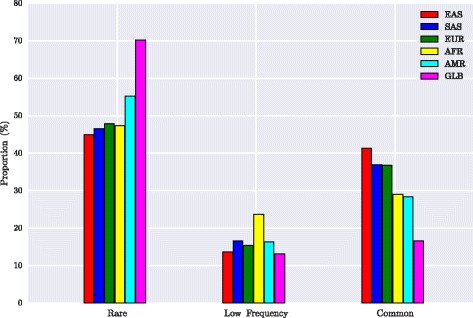
Frequency spectrum for 1 KG indels predicted as pathogenic. Comparison between non-coding variants across populations and stratified according to allele frequency (AF <1% for rare indels and AF >5% for common indels)

Exploiting AF data, it is possible to analyse how evolutionary pressures are acting outside the exome by considering the frequency spectrum of indels predicted as pathogenic. We examined the distribution of 1KG indels by binning variants into three categories (Fig. 3). Rare indels have AF<0.01, low frequency indels have AF∈ [0.01,0.05], whereas common indels have AF>0.05. Purifying selection removes disadvantageous alleles by reducing their frequency in a population. Therefore, common indels are less likely to be pathogenic than rare indels. We observed this phenomenon across all continental and global populations where the highest percentages of pathogenic indels are rare. Within the continental populations, AMR recorded the highest ratio (55%), followed by EUR (48%), AFR (47%), SAS (47%), and EAS (45%). This trend is even more prominent in the global population where the vast majority (70%) of pathogenic indels are rare. Non–rare variants shared across populations are typically older than non–rare private mutations and, therefore, less likely to be pathogenic.

Furthermore, by looking at common indels, we can analyse how bottlenecks have differentially effected populations. A drastic reduction in population size followed by a rapid growth enables deleterious variants to accumulate at high frequency [28]. European and Asian have been subjects of severe bottlenecks [27, 28] and, as can be seen in Fig. 3, these populations harbour higher ratios of pathogenic indels which are common. EAS has the highest percentage (41%) of disadvantageous common indels, followed by SAS (37%) and EUR (37%). Conversely, the African population is characterised by a much smaller ratio of pathogenic and common indels (29%). Interestingly, at least for the indels that we were able to score, the distribution of AMR indels is much more similar to the AFR frequency spectrum as, for instance, only 28% of pathogenic indels are common in the American population.

## Discussion

**Fig. 4:**
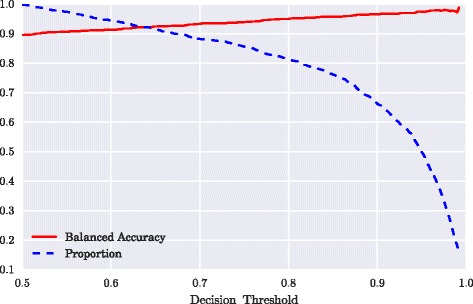
Cautious classification of benchmark indels. Balanced accuracy, over validation data, as a function of the decision threshold. By selecting only predictions with highest confidence, FATHMM-indel is capable of achieving almost perfect classification

We presented FATHMM–indel, an integrative method to assess mutation tolerance throughout the *whole* non–coding genome. When validated on benchmark data, FATHMM–indel outperformed CADD and GAVIN, state of the art models for predicting the functional impact of non–coding variants. In addition to predicting the functional class (pathogenic or neutral) of an indel, our method is capable of prioritising variants by computing a standardised score (*σ*) for each test mutation. This introduces an additional level of flexibility by enabling the implementation of cautious classification to only consider predictions with highest confidence. Given the distribution of FATHMM–indel scores over validation indels, it is possible to cautiously classify our benchmark data set. For example, one can predict an indel with a score bigger than the 80th percentile (0.967) as pathogenic, whilst a mutation with a *σ* smaller than the 20th percentile (0.034) as neutral. This restricts the number of variants classified to 40% of all benchmark indels but, crucially, allows FATHMM–indel to achieve almost perfect performance with a balanced accuracy of 98%. The interplay between balanced accuracy and the proportion of benchmark indels cautiously classified is comprehensively visualised in Fig. 4. Cautious classification could be extremely useful in, for instance, medical genetics research where, from a “pool” of putative variants, one is interested in selecting only a small subset of *candidate* mutations for experimental validation.

Given current estimates quantifying that *at least* 5% of the human genome is evolutionary constrained [29], it is crucial to deepen our understanding of how selective pressures are acting on non–coding elements. The distribution and evolution of deleterious alleles are fundamental in elucidating the genetic architecture of human disease. In this work, we have also shown how FATHMM–indel can be valuable to discover and analyse differences in non–coding mutation loads across populations.

Our model is available through a web server at *indels.biocompute.org.uk*. By uploading a file in (simplified) VCF, users can submit batches of indels. For a large submission of 10,000 variants, the web server returns FATHMM–indel scores within 30 min (on average).

FATHMM–indel was developed by harvesting knowledge from multiple genomic sources and performing integration at the level of data, where all features are annotated in one data set and similarities between examples are encoded in a unique kernel. As an avenue for future research, it would be interesting to investigate whether it is possible to further boost FATHMM–indel’s performance by implementing multiple kernel learning (MKL). Within an MKL approach, multiple data sources are arranged into several feature *groups*, each with its own kernel matrix – see, e.g [30]. Further data sources are available thanks to the efforts of projects like the encyclopedia of DNA elements (ENCODE) consortium [31], which also aims at mapping functional and regulatory elements located *outside* protein coding regions. For example, it would be possible to annotate an additional feature group from transcription factor binding sites data, which have recorded excellent predictive power in assessing genomic tolerance of non–coding SNVs [15].

Currently, as a consequence of our data collection protocol, FATHMM–indel is unable to accurately prioritise non–coding variants located in repetitive regions. Before all repeats were filtered from our training data, 1% of pathogenic indels were repeats whilst 21% of neutral indels were located in repetitive elements. Annotating a training set by random sampling of repetitive sequences would lead to over representation of repeats within the neutral class and, consequently, result in the introduction of a potential confounding factor. Hence, extending FATHMM–indel’s capabilities to prioritise repeats warrants further and careful analyses that we leave to future work.

## Conclusions

We developed FATHMM–indel, an integrative computational model for predicting indel pathogenicity. Although the vast majority of genetic alterations lie outside the exome, there is a lack of methods *specifically* designed to predict the impact of indels throughout the *whole* non–coding genome. We developed our model to fill in this gap, to aid in predicting the biological consequences of non–coding variants. We envisage FATHMM–indel as a useful annotation tool that could be used, for example, to prioritise causative variants, like those identified in GWASs, for downstream studies to analyse the phenotypic impact of non–coding indels.

## Additional file

Additional file 1 Supplementary Materials. In this PDF file, we report a detailed description of all the features used during the development of FATHMM-indel. (PDF 150 kb)

## Abbreviations

AF: Allele frequency
AFR: Africa
AMR: America
AUC: Area under the ROC curve
CADD: Combined annotation dependent depletion
EAS: East Asia
EUR: Europe
EVS: Exome variant server
FATHMM: Functional analysis through hidden Markov models
FN: False negative
GAVIN: Gene aware variant interpretation
GLB: Global
GWAS: Genome wide association study
HGMD: Human gene mutation database
Indel: Insertion or deletion
MAF: Minor allele frequency
MCC: Matthews correlation coefficient
NCV: Nested cross validation
ROC: Receiver operating characteristic
SAS: South Asia
SNV: Single nucleotide variant
SVM: Support vector machine
VCF: Variant call format
1KG: 1000 genomes
