# Supplementary material for: An integrative approach to predicting the functional effects of small indels in non-coding regions of the human genome
Source: BMC Bioinformatics. 2017 Oct 6;18:442. doi: 10.1186/s12859-017-1862-y (PMC5955213; doi:10.1186/s12859-017-1862-y)
Supplement: Supplementary file 1 — Supplementary Materials. In this PDF file, we report a detailed description of all the features used during the development of FATHMM-indel. (PDF 150 kb) [file 12859_2017_1862_MOESM1_ESM.pdf]

An Integrative Approach to Predicting the Functional Effects of Small Indels in Non–coding Regions of the Human Genome

Michael Ferlaino, Mark F. Rogers, Hashem A. Shihab, Matthew Mort, David N. Cooper, Tom R. Gaunt, and Colin Campbell

Supplementary Materials

1 FATHMM–indel's Features

Each indel in our data sets was annotated using DNA conservation data obtained from multiple sequence alignments (MSAs) of vertebrate genomes to the human genome [1]. The details on how covariates were computed are reported below. The features used are:

- P_r. This covariate represents FATHMM's emission probability for the reference nucleotide [2].
- P_m. FATHMM's emission probability for the mutant nucleotide [2].
- $P_m - P_r$ and $|P_m - P_r|$. These scores measure the potential impact of a mutation: the greater the difference, the greater the impact.
- MSA depth (the number of species used in MSAs). This feature is positively correlated with the confidence of the resulting conservation scores.
- FATHMM score. The log–odds ratio between P_r and P_m.
- PhyloP score [3]. PhyloP scores measure evolutionary conservation at individual alignment sites, ignoring neighbouring nucleotides in the calculation. It can be used to measure acceleration (evolution faster than expected under neutral drift) as well as conservation (evolution slower than expected).
- PhastCons score [4]. PhastCons scores account for regions surrounding each alignment position in estimating the probability that each nucleotide belongs to a conserved element.
- Percent identity. This value quantifies the similarity between wildtype and mutant sequences.

With the exception of percent identity, all covariates can be computed using data coming from MSAs between human and 45 vertebrate genomes (8 "46 way" features), or using MSA data between human and 99 vertebrate genomes (8 "100 way" features). In this work we considered both "46 way" and "100 way" covariates, culminating in a total of 17 $(8 + 8 + 1)$ features (the study was performed using the human genome assembly GRCh37).

1.1 Computation of Conservation Scores

To illustrate how single–nucleotide features are integrated into an indel score, let us consider a three–nucleotide reference sequence `AGC`. A two–nucleotide insertion `TT` starting at the second position will yield the mutant sequence `ATTGC`. The mutation's overall conservation score includes the score for the first nucleotide, `A`, along with the scores from the inserted nucleotides `TT`. We compute the effect of a transition from the three–nucleotide wildtype sequence `AGC` to the three–nucleotide mutant sequence `ATT` by averaging the conversion scores for three transitions: $\texttt{A} \rightarrow \texttt{A}$, $\texttt{G} \rightarrow \texttt{T}$, and $\texttt{C} \rightarrow \texttt{T}$. The approach for deletions is similar: we simply average the scores for mutations along the length of a deletion.

These mean scores provide a simple way to assess conservation across an indel. In coding regions, even a single–nucleotide insertion/deletion can impact many positions downstream. In non–coding regions, indels may disrupt binding by altering recognition motifs or by changing the relative positions of motifs. More sophisticated scoring procedures may thus be warranted, but we found that this simple procedure appears to yield highly informative features for our model.

When methods provide p values (*e.g.* PhastCons), the indel score is simply the average of the p values. However, it does not always make sense to take a simple average for scores from other methods.

For example, FATHMM [2] scores f_{SNV} are (logarithmic) ratios based on probabilities for reference (P_r) and mutant (P_m) nucleotides:

$$f_{\mathrm{SNV}} = \ln \frac{P_r(1-P_r)^{-1}}{P_m(1-P_m)^{-1}}$$

To compute a score over a range, we compute the average reference probability ($\bar{P}_r$) and the average mutant probability ($\bar{P}_m$) across the range and then compute a ratio from these average probabilities:

$$f_{\mathrm{indel}} = \ln \frac{\bar{P}_r(1-\bar{P}_r)^{-1}}{\bar{P}_m(1-\bar{P}_m)^{-1}}$$

PhyloP also requires special handling. Briefly, these scores may be split into two categories based on positive or negative values. Positive values indicate whether a position is unusually highly conserved, even if it falls within a conserved region. Negative values indicate whether a position is unusually variable, even within a variable region. Either a positive or negative PhyloP score S_{phy} may be converted into a probability P_e that the value is extreme using:

$$P_e = 10^{-|S_{\mathrm{phy}}|}$$

By using $|S_{\mathrm{phy}}|$ we guarantee that $P_e \in [0, 1]$. To compute a score that reflects conservation characteristics across a region, we convert scores to probabilities for positive and negative scores, then subtract the probabilities associated with negative scores from those associated with positive scores to obtain a total. We then divide this by the number of positions to get an average for the region. These averages fall into the range $[-1, +1]$ and thus correspond to sequence changes that range from least (-1) to most ($+1$) disruptive.

1.2 Percent Identity Scores

Although we eliminated repeat sequences from our data, some indels are likely to be more disruptive than others – by changing local GC content, for example. As a simple way to encapsulate the disruption an indel may impart to a genomic region, we included a percent identity feature to compare the wildtype and mutant sequences. To compute this feature we consider the region around an indel's starting position s and compare the reference sequence R with the mutated sequence M that results from the indel. For an indel of length k, R is the reference genomic sequence from s to $s+k$. For an insertion, the mutant sequence M is simply the annotated insert sequence. For a deletion of length k, we construct M from the reference sequence starting at position $s+k$ and ending at $s+2k$. In each case we compute the percent identity f_{PID} between R and M as follows:

$$f_{\mathrm{PID}}(R, M) = \frac{1}{k} \sum_{i=1}^{k} I(R_i = M_i)$$

where I is the indicator function.

References

[1] Blanchette, M., Kent, W.J., Riemer, C., Elnitski, L., Smit, A.F., Roskin, K.M., Baertsch, R., Rosenbloom, K., Clawson, H., Green, E.D., Haussler, D., Miller, W.: Aligning multiple genomic sequences with the threaded blockset aligner. Genome Res **14**, 708–715 (2004)

[2] Shihab, H.A., Gough, J., Cooper, D.N., Stenson, P.D., Barker, G.L., Edwards, K.J., Day, I.N., Gaunt, T.R.: Predicting the functional, molecular, and phenotypic consequences of amino acid substitutions using hidden markov models. Hum Mutat **34**, 57–65 (2013)

[3] Pollard, K.S., Hubisz, M.J., Rosenbloom, K.R., Siepel, A.: Detection of nonneutral substitution rates on mammalian phylogenies. Genome Res **20**, 110–121 (2010)

[4] Siepel, A., Bejerano, G., Pedersen, J.S., Hinrichs, A.S., Hou, M., Rosenbloom, K., Clawson, H., Spieth, J., Hillier, L.W., Richards, S., Weinstock, G.M., Wilson, R.K., Gibbs, R.A., Kent, W.J., Miller, W., Haussler, D.: Evolutionarily conserved elements in vertebrate, insect, worm, and yeast genomes. Genom Res **15**, 1034–1050 (2005)
